# TikTok as a source of information regarding premature ejaculation: a qualitative assessment

**DOI:** 10.1093/sexmed/qfac020

**Published:** 2023-03-01

**Authors:** Ari Bernstein, Michael Zhu, Justin Loloi, Mustufa Babar, Nick Winokur, Matthew Wysocki, Seth Cohen

**Affiliations:** Department of Urology, New York University Langone Health, New York, NY, United States; Albert Einstein College of Medicine, Bronx, NY, United States; Montefiore Medical Center, Department of Urology, Bronx, NY, United States; Albert Einstein College of Medicine, Bronx, NY, United States; Albert Einstein College of Medicine, Bronx, NY, United States; Albert Einstein College of Medicine, Bronx, NY, United States; Department of Urology, New York University Langone Health, New York, NY, United States

**Keywords:** premature ejaculation, disorders of ejaculation, sexual medicine, andrology

## Abstract

**Background:**

Patients are increasingly looking to social media platforms for medical information.

**Aim:**

In this study we aimed to evaluate the quality of information regarding premature ejaculation (PE) on TikTok.

**Methods:**

The term “premature ejaculation” was searched on TikTok on a single day in May 2022. Videos were sorted by 3 reviewers as reliable or unreliable based on the accuracy of video content. Relevant user metrics were collected for each video, including the numbers of likes, shares, and followers, and the video length, source of upload, and speaker type. The quality of information was objectified with 2 validated tools, with mean scores obtained from the 3 reviewers, the Patient Education Materials Assessment Tool (PEMAT) and the 5-point modified DISCERN instrument.

**Outcomes:**

Outcomes were video reliability categorization, video and user metrics as described above, and video quality as quantified by PEMAT and DISCERN scores.

**Results:**

Eight videos were categorized as reliable and 32 videos were categorized as unreliable. The mean number of “likes” per video was higher in the reliable than in the unreliable group (1238 vs 126, *P* < .018). Accounts posting reliable videos had higher mean numbers of followers than those posting unreliable videos (55 050 vs 12 042, *P* = .025). The majority of unreliable videos (75%) vs reliable videos (12.5%) were posted by self-identified patients or individual users, whereas 62.5% of reliable videos vs versus 6.3% of unreliable videos were posted by individual physicians or physician groups. Few videos overall mentioned PE definition, indications for PE treatment, types of treatment, or value of psychological intervention (12.5%, 15%, 22.5%, and 5.0% of videos, respectively). Video length and number of shares did not differ between groups. Reliable videos had higher PEMAT (73.0 vs 45.1, *P* < .001) and DISCERN (2.7 vs 0, *P* < .001.) scores.

**Clinical implications:**

There exists a critical need for enhanced quality of medical information on social media platforms in hopes of encouraging patients with impaired sexual function to seek appropriate medical care.

**Strength and limitations:**

Strengths of this study include the objective use of validated quality assessment tools and a focus on TikTok as an emerging social media platform. Limitations include large numbers of excluded videos.

**Conclusion:**

The quality of available information regarding PE on TikTok is low, with a significant percentage of videos on this topic fraught with inaccuracies. Given TikTok’s prominence as a social media platform primarily geared toward younger audiences, we emphasize the need for improvement in the quality of information available regarding PE and its management.

## Introduction

Premature ejaculation (PE) is one of the most common disorders of male sexual function. Despite a prevalence of up to 30%, PE remains underdiscussed and undertreated, with an estimated 9% of patients with clinically significant PE ultimately seeking professional medical advice.^1^ Among younger patients, the prevalence of PE remains similar, hovering around 20%-25%.^2^ These epidemiological numbers, however, are based on single-item questionnaires that do not account for chronicity of symptoms or level of bother experienced by both the self and partner. It has been theorized that the true prevalence of clinically bothersome PE is far lower than previously suggested, likely around 5%.^3^ Cited underlying reasons for avoiding medical help include a general lack of awareness about PE, stigma associated with its diagnosis, embarrassment, helplessness, and a problematic reluctance to discuss male sexual health with physicians.^4^^,^^5^ As a result of these barriers, many patients with impaired sexual functioning secondary to PE remain undertreated.^6^

Barriers to proper medical attention for PE by sexual medicine specialists lead patients to seek out less confrontational methods of obtaining medical advice, such as turning to social media platforms. Approximately 60% of adults in the United States have reported looking for medical information online.^7^ TikTok, a relatively new video-based platform founded in 2016, has become one of the most popular social media platforms among a younger audience, currently with 800 million active users and more than 1 billion videos viewed daily.^8^ TikTok has become especially popular among patients 13 to 24 years old, a population that is both sexually active and also burdened with high rates of mental health issues that often go unaddressed into adulthood.^9-12^ TikTok’s short-length videos and bite-sized content provide quick and manageable opportunities for patient education across all medical sectors, with great potential for providing medical and public health information to wide audiences.^13^

Although medical misinformation is reportedly prohibited on TikTok according to community guidelines, a recent systematic review found that the prevalence of misinformation was high across the majority of social media platforms.^14^ Given TikTok’s recent entrance into the social media landscape, there is little research on the quality and characteristics of its medical content. While studies have evaluated the quality of content surrounding PE and its treatments on YouTube, another popular video-based social media platform, to our knowledge there have been no studies to date evaluating the quality and accuracy of PE information quality on TikTok.^6^^,^^15^ In this study we aimed to evaluate and describe the current landscape of information regarding PE on TikTok, with emphasis on content quality and reliability.

## Materials and methods

**Figure 1:**
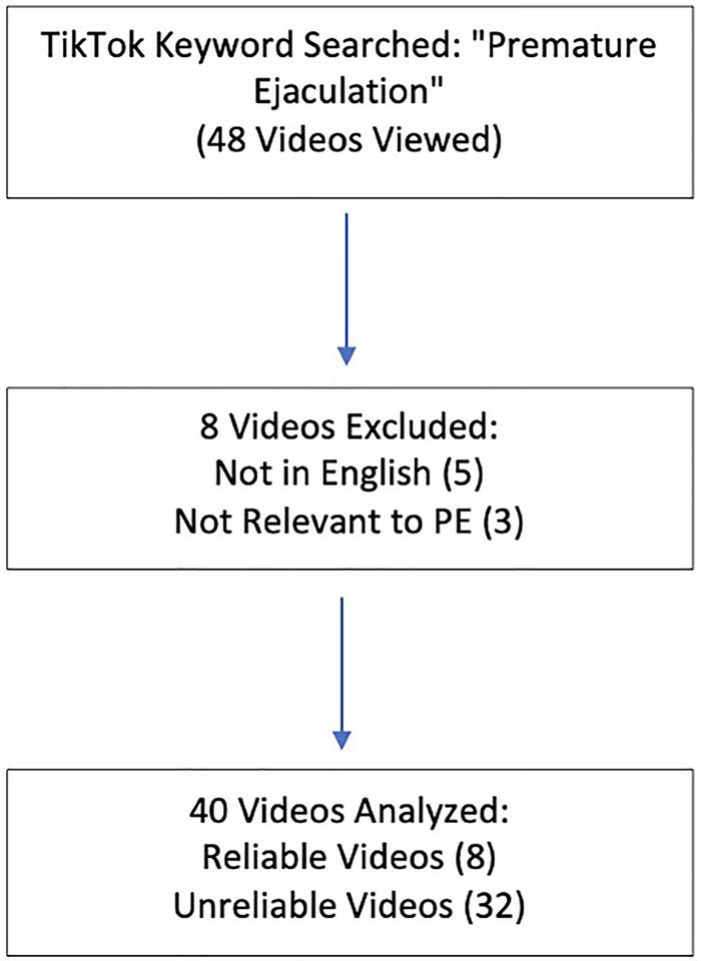
Flowchart of TikTok video selection.

The term “premature ejaculation” was searched on TikTok on May 16, 2022, on the mobile application without logging into a personal account. The selected videos were based on the “top” videos on the selected date of query and were included if they contained content relevant to PE. Of note, similar teams such as “rapid ejaculation” and “premature orgasm” were queried with near zero yield and were excluded from this study. Videos were excluded if they contained content that was not relevant to PE, had no accompanying audio or subtitles, or if they had narration and/or text in a language other than English. The first 40 videos that met inclusion criteria were included in the study (Figure 1).

Relevant user metrics were collected for each video, including video length, number of likes, number of shares, number of followers for the account posting the video, date of original post, source of upload (individual user/patient, physician/physician group, nonphysician, nonphysician group, or medical advertisement/for-profit company) and speaker type (individual user/patient, physician, nonphysician healthcare provider, unidentified narrator, or no speaker). Additionally, mention of PE, mention of indications for treatment, mention of epidemiological factors (incidence/prevalence) of condition, mention of behavioral techniques and/or medication, and mention of psychological treatment/resources for patients suffering from condition were all collected.

To evaluate the quality of information regarding PE on TikTok, the videos were classified into one of the following two groups, reliable or unreliable, based on the accuracy of the information presented.

### Reliable information

The TikTok videos were classified as reliable if they contained medically accurate information about the disease and/or options for treatment. Medically correct information about PE included, as per the American Urological Association (AUA), the definition, indications for treatment, and/or epidemiological factors. To be considered valid, definitions for PE had to specifically included mention of any of the 3 components of PE: short latency period, lack of control of ejaculation, and clinically significant bother associated with ejaculation. Reliable AUA-recommended treatment options included psychological/behavioral strategies (such as the stop and start program, squeeze technique, or sensate focus masturbation before sexual intercourse), pharmacological options (including dapoxetine, other off-label antidepressants, topical anesthetic agents, tramadol, phosphodiesterase-5 inhibitor [PDE5i], or combination treatments) or other scientifically proven methods (acupuncture or modanafil).

### Unreliable information

The TikTok videos were classified as unreliable if they contained medically inaccurate information about PE or about treatment modalities that were not supported by research or recommended by current guidelines. Medically inaccurate information included unsupported claims about treating PE with herbal medicine, breathing techniques, or anything else that is not supported in the literature. If a video contained both unreliable and reliable information, it was classified as unreliable. In the event of a discrepancy, consensus arbitrated the disagreement.

Two validated scoring tools, DISCERN and the Patient Education Materials Assessment Tool (PEMAT), were used to evaluate the quality of information provided in the videos. DISCERN is a 15-question tool, with each question rated on a 5-point scale, designed to help users of consumer health information judge the quality of information about treatment options.^16^ For this study, a modified 5-question DISCERN tool, with each question scored on a 5-point scale, was used to judge the reliability of information, along with PEMAT, an instrument designed to assess the understandability and actionability of both print and audiovisual patient education materials.^17^ In this study we used the audiovisual version of PEMAT, which consists of 13 items measuring understandability and 4 items measuring actionability.

Three qualified reviewers with formal urologic training collected the data and evaluated the videos using DISCERN and PEMAT. The mean scores for DISCERN and PEMAT were calculated based on the scores from the 3 reviewers. In the event of a discrepancy, consensus arbitrated the disagreement.

## Results

A total of 40 videos on TikTok were included in the analysis. The numbers of likes and shares, number of followers, video length, source of upload, speaker type, and narrator mentions of PE definition, treatment behavioral techniques, and psychological resources are included in Table 1. The majority of videos (62.5%) were posted by an individual user/patient, with physician/physician group comprising only 17% of uploads. There was a high representation of individual user/patient speakers (45.0%) in the videos. Few videos overall mentioned PE definition, indications for PE treatment, types of treatment, or value of psychological intervention (12.5%, 15%, 22.5%, and 5.0% respectively).

**Table 1:** Video characteristics by accuracy.

	**All videos**	**Reliable**	**Unreliable**	***P* value**
No. of videos	40	8	32	
Likes	255 (41-2085)	1238 (507-7846)	126 (30-1043)	.018
Shares	22 (2-125)	79 (19-117)	16 (1-154)	0.12
No. of followers	18 600 (3102-77 300)	55 050 (20 250-199 050)	12 042 (942-42 825)	.025
Video length in seconds	22 (12-59)	45 (14-58)	21 (9-60)	0.54
Source of upload				<.001
Individual user/patient	25 (62.5)	1 (12.5)	24 (75.0)	
Physician/physician group	7 (17.0)	5 (62.5)	2 (6.3)	
Nonphysician/nonphysician group	3 (7.5)	2 (25.0)	1 (3.1)	
Medical advertisement/for-profit company	5 (12.5)	0 (0)	5 (15.6)	
Speaker				<.001
Individual user/patient	18 (45.0)	1 (12.5)	17 (53.1)	
Physician	7 (17.5)	5 (62.5)	2 (6.3)	
Nonphysician healthcare provider	3 (7.5)	2 (25.0)	1 (3.1)	
Unidentified narrator	2 (5.0)	0 (0)	2 (6.3)	
No speaker	10 (25.0)	0 (0)	10 (31.3)	
Narrator mentions				
Premature ejaculation definition	5 (12.5)	3 (37.5)	2 (6.3)	.017
Indications for treatment	6 (15.0)	2 (25.0)	4 (12.5)	.376
Behavioral techniques and/or medication	9 (22.5)	6 (75.0)	3 (9.4)	<.001
Psychological treatment/resources	2 (5.0)	1 (12.5)	1 (3.1)	.277

Of the 40 videos, eight videos were categorized as reliable, and 32 videos were categorized as unreliable (Table 1). The mean number of “likes” was higher in the reliable group than the unreliable group (1238 vs 126, *P* < .018). Accounts posting reliable videos had higher mean numbers of followers than the accounts posting unreliable videos (55 050 vs 12 042, *P* = .025). There were no significant difference in number of shares or video length between the 2 groups.

There were significant differences in the sources of the upload between the reliable videos and the unreliable videos (Table 1). The reliable videos had a greater proportion of posts by individual physicians or physician groups compared to the unreliable videos (62.5% vs 6.3%, *P* < .001). The majority of unreliable videos were posted by self-identified patients or individual users, with few reliable videos posted by self-identified patients or individual users (75% vs 12.5%, *P* < .001). No reliable videos were posted as medical advertisements or by for-profit-companies, compared to 15.6% of unreliable videos.

There were significant differences in the speaker type between the reliable videos and the unreliable videos (Table 1). Reliable videos had a higher representation of physician speakers compared to unreliable videos (62.5% vs 6.3%, *P* < .001). Unreliable videos had a higher representation of individual user/patient speakers (53.1% vs 12.5%, *P* < .001), unidentified narrator (6.3% vs 0%, *P* < .001), or no speaker (31.3% vs 0%, *P* < .001) than reliable videos.

While few videos mentioned indications for PE treatment, types of treatment, or value of psychological intervention, significant differences were observed when the reliable videos were compared with the unreliable videos (Table 1). A higher percentage of reliable videos defined PE compared to unreliable videos (37.5% vs 6.3%, *P* = .017). Reliable videos were more likely to mention behavioral interventions than unreliable videos (75% vs 9.4%, *P* < .001). There were no significant differences in mentions of indications for treatment or psychological treatment/resources between the two groups of videos.

There were significant differences in the DISCERN and PEMAT scores between the reliable and unreliable videos (Table 2). Reliable videos had a higher DISCERN score (2.7 vs 0, *P* < .001) than the unreliable videos. In addition, the reliable videos had a higher PEMAT understandability percentage (86.3% vs 54.2%, *P* < .001), a higher PEMAT accountability percentage (72.2% vs 16.7%, *P* = .045), and a higher PEMAT total percentage (73.0% vs 45.1%, *P* < .001) than the unreliable videos.

**Table 2:** DISCERN and PEMAT Scores for all videos and grouped by reliable and unreliable videos.

	**All videos**	**Reliable**	**Unreliable**	***P* value**
DISCERN score	0.5 (0-1.7)	2.7 (1.8-3.8)	0 (0-0.7)	<.001
PEMAT understandability, %	58.1 (47.6-68.1)	86.3 (67.9-93.7)	54.2 (42.7-62.3)	<.001
PEMAT accountability, %	44.4 (0-77.8)	72.2 (44.4-88.9)	16.7 (0-66.7)	.045
PEMAT total, %	50.5 (39.4-70.1)	73.0 (69.9-83.3)	45.1 (36.1-59.6)	<.001

PEMAT, Patient Education Materials Assessment Tool.

## Discussion

This study is to our knowledge the first to investigate the quality and content of PE videos on TikTok, an emerging social media platform that has attracted a large userbase, primarily among younger audiences.^10^ Given that research has shown that many young people between the ages of 18 and 30 years rely on online sources for medical information, it is vital to investigate the quality of health content on TikTok and find ways to combat the spread of health misinformation.^18^ In addition, enhancing the quality of medical information on TikTok and other social media platforms is of great importance because studies have shown young adults to have relatively low levels of health literacy despite high levels of digital literacy.^19^ Despite TikTok’s growing popularity, less than 180 articles on the PubMed database reference the application, and even fewer discuss the platform’s content regarding urological conditions.^20^ Previous studies have examined the quality and characteristics of TikTok videos regarding prostate cancer, genitourinary cancers, pediatric urological issues, and erectile dysfunction;^21-23^ however, none have evaluated the quality and content of PE videos specifically.

Our study revealed that the majority of TikTok videos discussing PE were unreliable both in our own assessment and according to validated quality assessment tools (DISCERN and PEMAT). This result was not unexpected; a recent study by Xu et al. found that 98% of the videos they had assessed on TikTok regarding prostate cancer were moderate to poor quality, and 47% of the videos containing objective information had a significant amount of misinformation.^7^ A recent study by Kaynak et al. found that PE content on YouTube was riddled with inaccuracies and generally of low quality.^24^ A large analysis of men’s health content across TikTok and Instagram by Dubin et al. similarly found consistently low quality of sexual health content across social media platforms.^25^ Similar to a recent study comparing quality of content addressing erectile dysfunction between TikTok and YouTube, a small minority of videos were posted by physicians/physician groups, which could speak to either the large number of non–medical professional videos being posted or to a broader lack of attention that physicians give to providing sound medical information on social media platforms.^23^ Further research is needed to characterize the behavior of physicians and their contributions of content to social media platforms for patient consumption.

Despite the small proportion of physician-generated content on TikTok, we found that physician-generated videos were much more likely to include reliable information than other individual/patient content. We also found that reliable videos had significantly higher engagement and the accounts creating those videos had more followers, demonstrating that users seem to recognize and respond positively to higher levels of information quality and overall content reliability. Great potential exists for physicians and physician groups to capitalize on this finding and to contribute high-quality content, promote accurate medical information, and guide patients toward appropriate medical attention and treatment. Our study findings are in accordance with findings of other studies demonstrating the high quality of physician-generated content. Om et al. found that DISCERN scores were significantly higher among videos produced by physicians discussing aesthetic surgical procedures than videos by any other individuals.^26^ Yeung et al. reported that when searching for content regarding ADHD on TikTok, healthcare providers uploaded higher quality and more useful videos than non–healthcare providers.^27^ As the potential for widespread dissemination of health misinformation on TikTok grows, physicians become more responsible for leveling the playing field and creating quality information in an effort to combat misinformation that targets a particularly vulnerable young patient population.

In this study we found that few videos mentioned indications for PE treatment, types of treatment, or the value of psychological intervention, and that even videos deemed reliable did not address those topics, similarly to the unreliable videos. However, reliable videos discussed behavioral techniques, such as the squeeze and stop-start techniques, significantly more than unreliable videos. We believe that this difference is due to these behavioral interventions being ones that users can attempt at home without much professional guidance, perhaps what users are looking for on social media platforms. Alternatively, this emphasis on behavioral methods may be attributable to the fact that any reference to medications could necessitate a discussion about mechanism of action, cost, and side-effects—a discussion far lengthier than the bite-sized style featured on TikTok. Interestingly, we noticed that many videos promoted alternative or holistic treatments that relied on natural ingredients. Physicians would benefit from familiarizing themselves with popularized, nontraditional treatments that patients may want to discuss.

There are some limitations to this study. The strict provisional diagnoses of Waldinger and Schweitzer (ie, natural variable and subjective PE) were not utilized in our search query.^28^ The sample size is relatively small as it only included 40 videos. Evaluations of TikTok videos using DISCERN and PEMAT are subject to observer bias; however, our high positive kappa coefficient and intraclass correlation coefficients demonstrated strong interobserver reliability. As a cross-sectional analysis, in our study we surveyed only the top 40 videos, which were found by using the TikTok search algorithm for “top” videos. Less popular videos may have different qualities and characteristics that are not captured in this analysis. Similarly, we used an English language search query, “premature ejaculation.” Consequently, videos that may discuss PE but may be found in a different language would not have been discovered. Our review of the videos did not specifically evaluate for the breakdown of which videos specifically discussed lifelong vs acquired PE, although any medically accurate mention of either PE type was incorporated into the designation as reliable or unreliable. The provisional diagnoses of Waldinger and Schweitzer (ie, natural variable PE and subjective PE) were not accounted for in this study.

Despite these limitations, we believe the results of this study accurately reflect the current state of information being disseminated through TikTok regarding PE. Given the sensitive nature of sexual health, patients with PE may hesitate to seek professional medical advice from physicians and instead turn to the internet. It is therefore important for clinicians to be aware of the spread of misleading information, especially from social media platforms such as TikTok. Simultaneously, it is becoming more important for the medical community to collaborate and create informed and understandable content, especially on younger platforms like TikTok. A stronger understanding of the nature of misinformation in other sensitive sexual health topics may illuminate broader trends of health misinformation on social media. Further studies to determine whether increased physician engagement on social media platforms can correct misinformation are warranted.

## Conclusion

The quality of information regarding PE on TikTok, a prominent social media platform geared toward younger audiences, is low. Existing video content on TikTok regarding PE is fraught with medically inaccurate information being shared by sources with little credibility or expertise. We emphasize a need for improvement in the quality of information available on social media platforms regarding PE and its management to help combat barriers to appropriate medical information.

## Funding

None declared.

*Conflicts of interest:* None declared.
